# Community perspectives and experiences of quality maternal and newborn care in East New Britain, Papua New Guinea

**DOI:** 10.1186/s12913-023-09723-x

**Published:** 2023-07-20

**Authors:** Alyce N. Wilson, Pele Melepia, Rose Suruka, Priscah Hezeri, Dukduk Kabiu, Delly Babona, Pinip Wapi, Alison Morgan, Joshua P. Vogel, James Beeson, Christopher Morgan, Angela Kelly-Hanku, Michelle J. L. Scoullar, Somu Nosi, Lisa M. Vallely, Elissa Kennedy, Meghan A. Bohren, Caroline S. E. Homer

**Affiliations:** 1 Maternal, Child and Adolescent Health Program, Burnet Institute, Melbourne, Australia; 2 School of Population and Global Health, Nossal Institute for Global Health, University of Melbourne, Melbourne, Australia; 3 Healthy Mothers, Healthy Babies, Burnet Institute, Kokopo, Papua New Guinea; 4 St Mary’s Hospital, Kokopo, Papua New Guinea; 5 Nonga General Hospital, Rabaul, Papua New Guinea; 6 Global Financing Facility, World Bank, Washington, DC USA; 7 Department of Medicine, University of Melbourne, Melbourne, Australia; 8 Immunization Program, JHPIEGO, Johns Hopkins University, Baltimore, USA; 9 Papua New Guinea Institute for Medical Research, Goroka, Papua New Guinea; 10 Kirby Institute, University of New South Wales, Kensington, Australia; 11 Gender and Women’s Health Unit, School of Population and Global Health, Centre for Health Equity, University of Melbourne, Melbourne, Australia

**Keywords:** Quality Care, Maternal and Newborn Health, Papua New Guinea, Quality Improvement, Community

## Abstract

**Background:**

Quality maternal and newborn care is essential for improving the health of mothers and babies. Low- and middle-income countries, such as Papua New Guinea (PNG), face many barriers to achieving quality care for all. Efforts to improve the quality of maternal and newborn care must involve community in the design, implementation, and evaluation of initiatives to ensure that interventions are appropriate and relevant for the target community. We aimed to describe community members’ perspectives and experiences of maternal and newborn care, and their ideas for improvement in one province, East New Britain, in PNG.

**Methods:**

We undertook a qualitative descriptive study in partnership with and alongside five local health facilities, health care workers and community members, using a Partnership Defined Quality Approach. We conducted ten focus group discussions with 68 community members (identified through church, market and other community-based groups) in East New Britain PNG to explore perspectives and experiences of maternal and newborn care, identify enablers and barriers to quality care and interventions to improve care. Discussions were transcribed verbatim. A mixed inductive and deductive analysis was conducted including application of the World Health Organisation (WHO) Quality Maternal and Newborn Care framework.

**Results:**

Using the WHO framework, we present the findings in accordance with the five experience of care domains. We found that the community reported multiple challenges in accessing care and facilities were described as under-staffed and under resourced. Community members emphasised the importance of good communication and competent, caring and respectful healthcare workers. Both women and men expressed a strong desire for companionship during labor and birth. Several changes were suggested by the community that could immediately improve the quality of care.

**Conclusions:**

Community perspectives and experiences are critical for informing effective and sustainable interventions to improve the quality of maternal and newborn care and increasing facility-based births in PNG. A greater understanding of the care experience as a key component of quality care is needed and any quality improvement initiatives must include the user experience as a key outcome measure.

**Supplementary Information:**

The online version contains supplementary material available at 10.1186/s12913-023-09723-x.

## Abstract (plain language summary)

Improving the care provided to, and experienced by, women and their families during pregnancy and childbirth is important for improving the health of mothers and babies. Community members should be involved in thinking about appropriate ways to improve care. Papua New Guinea (PNG) is a country in the Pacific which faces multiple challenges to improving care during pregnancy and birth. We aimed to understand what community members think about care provided and experienced during labour and birth in East New Britain, a rural province of PNG.

We worked with five health facilities, health workers and community members in East New Britain to develop a qualitative research project. We carried out 10 focus group discussions with community members in East New Britain to understand what the provision and experience of care was like during labour and birth, and ways that it could be improved.

We found that community members identified multiple challenges in getting to facilities and many facilities were found to have not enough supplies, equipment, or staff. Community members wanted staff that were good at their work but also caring and respectful. Women wanted to have support people present during labour and birth and many men wanted to be present too.

Our results show that it is important to understand what the community thinks about the quality of care during labour and birth and this information is helpful to design effective activities to improve the care provided and experienced.

**Supplementary Information:**

The online version contains supplementary material available at 10.1186/s12913-023-09723-x.

## Background

Quality maternal and newborn care is essential for improving the health of mothers and babies. Quality care is characterized as care which is safe, effective, timely, efficient, equitable and person-centered [1, 2]. With respect to maternal and newborn care, quality care includes the provision of evidence-based care by skilled providers during pregnancy and birth in a respectful and supportive environment [3]. Low- and middle-income countries (LMICs), such as Papua New Guinea (PNG), face many barriers to achieving quality care for all [4]. PNG is a lower-middle income country in the Pacific region with a population of over 10 million people, mostly (85%) in rural areas [5]. It has some of the poorest maternal and newborn health indicators in the Pacific region [6]. Improving the quality of maternal and newborn care in PNG is critical to addressing the high rates of maternal and newborn morbidity and mortality [7, 8].

The Maternal Mortality Ratio in PNG is one of the highest in the Pacific region [9]; estimates for PNG vary (215 to 930 per 100,000 live births), but the broadly accepted figure is around 500 maternal deaths per 100,000 live births [10]. Neonatal mortality is high, with a reported rate of 22 per 1000 live births [11], though this is likely under-estimated. The national stillbirth rate is similarly high, affecting up to 30 babies per 1,000 births [6]. These indicators are in part driven by low rates of antenatal care and facility-based births nationwide: only 54% of women receive at least one antenatal visit and on average 40% of women give birth in a facility [12]. These statistics are underpinned and compounded by high rates of unintended pregnancies, low modern contraceptive use [13], and a high burden of reproductive tract infections [14] which are associated with poor maternal and newborn health outcomes [15, 16].

Whilst quality of care encompasses both the provision and experience of care, there has been little emphasis on measuring the care experience and an under appreciation of why care experiences and patient-centered care matters in the perinatal literature [17, 18]. In 2016, the World Health Organization (WHO) published the *Standards for improving the quality of maternal and newborn care in health facilities framework* consisting of eight domains required for the provision and experience of quality maternal and newborn care. Three of these domains specifically related to the care experience – ‘effective communication’, ‘respect and dignity’, ‘emotional support’, and two co-relate to care provision and experience – ‘competent and motivated human resources’ and ‘essential physical resources available’ [3]. In 2019, the White Ribbon Alliance surveyed 1.2 million women from 114 countries about their needs for quality maternal and newborn care through their ‘What Women Want Campaign’ [19]. Key findings included women’s desire to be treated with respect and compassion, with access to clean, functioning, well-equipped health facilities. Global initiatives to improve the quality of maternal and newborn care, such as the Quality, Equity, Dignity Network [20] and Early Essential Newborn Care program [21], emphasise the need for community engagement and leadership in the design, implementation, and evaluation of quality improvement initiatives, to ensure that interventions are appropriate and relevant for the target community [22].

In PNG and the Pacific more broadly, there has been limited research on how to improve the quality of maternal and newborn care [4], particularly from the perspectives of community members [4, 7, 23]. The aim of this study was to focus on the care experience and describe women and men’s perspectives and experiences of maternal and newborn care and their ideas for improvement in East New Britain, PNG.

## Methods

### Study design

**Fig. 1:**
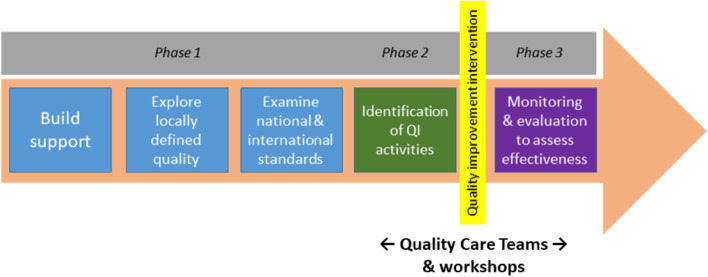
Partnership Defined Quality approach

This qualitative participatory descriptive study [24] is nested within a larger mixed-methods quality improvement study in East New Britain Province, PNG. The ‘Gutpela Helt Sevis Stadi’ (in English: ‘Quality Health Services Study’) involves working in partnership with and alongside local health facilities, healthcare workers and community members, to identify feasible, low-cost and effective quality improvement interventions to improve maternal and newborn health. The study was informed by Partnership Defined Quality (PDQ) (Fig. 1)—a co-design, participatory approach which has been used in the development sector to monitor and improve the quality of health services [25]. The PDQ approach was ideal for this project as it acts to optimise local community engagement by bridging quality assessment and technical improvement with community mobilisation through a four-step process: building support, exploring quality, bridging the gap, and working in partnership.

The PDQ approach facilitates the engagement of health management, healthcare workers and community members to explore individual and group perspectives of quality, develop solutions, and mutually commit to improve, implement and monitor quality improvement activities. Ethical approval was granted by relevant authorities in PNG and Australia (MRAC 19.16 and Project No. 267/19), and we have reported this work according to the consolidated criteria for reporting qualitative research (COREQ) checklist (Appendix 1) [26].

### East New Britain, Papua New Guinea

East New Britain is a rural province in the New Guinea Islands region of PNG, with a population of around 400,000 [27]. There are three main cultural groups in the province—the Tolai, Pomio and Baining people. English and Tok Pisin are official languages in PNG, however there are over 800 languages (Tok Ples) spoken. Whilst there is relatively good road access to towns and villages, the mountainous interior renders some communities accessible only by walking tracks, and many coastal villages are generally only accessible by boat [28]. As is the case in other regions and provinces in PNG, there is a chronic shortage of healthcare workers in East New Britain with only 15 healthcare workers per 10,000 population [29], well below WHO health workforce density recommendations of 44.5 per 10,000 population [30]. The health workforce is insufficient to meet the demand for maternal and newborn care, with approximately 10,000 babies born in East New Britain each year [31]. Approximately 70% of pregnant women in East New Britain attend at least one antenatal care visit compared to 50% nationally [29]. Rates of facility birth are also generally higher in East New Britain at 60%, compared to the national average of 40% [29]. Recent studies in this population have shown high rates of unintended pregnancy and low rates of modern contraceptive use [13], high rates of reproductive tract infections [14], and gaps in childhood immunisation coverage [32].

East New Britain has one tertiary hospital (Nonga General Hospital), one secondary hospital (St Mary’s/Vunapope Hospital), three rural hospitals, 32 health centers and 109 community health posts. Whilst all facilities are the responsibility of the Provincial Health Authority (PHA), Catholic and other faith-based health services manage approximately 50% of the health services in the province. A range of cadres make up the health workforce in PNG including doctors, nurses, midwives, Community Health Workers (CHWs) and Health Extension Officers (HEOs). In PNG, CHWs form part of the formal health system and complete two years of training; whilst HEOs are highly skilled clinicians with four years of training to manage rural and remote services. Tertiary hospitals are generally staffed by all cadres whilst community health posts may have one to two healthcare workers, often a CHW and a nurse. The National Department of Health expects all health facilities to be able to provide, at a minimum, basic emergency obstetric and newborn care [33] – however, this is often not feasible for understaffed, underequipped health facilities, especially in rural and remote areas.

### Study setting

This study was conducted in the catchment areas of five health facilities that provide maternity services in East New Britain: a tertiary referral hospital, two rural hospitals, one health centre and one community health post (Table 1). The PHA nominated these facilities to participate in the study because they collectively provide approximately 70% of the facility-based birthing services in the province, have been involved in previous research [13, 14, 32], represent a combination of Government- and faith-based organised services, and capture the referral pathway from a remote community health post to a tertiary referral hospital.

**Table 1:** Facilities

Description of facilities			
Facilities	**Average number of births/month**	**Maternity workforce**	CEmONC or BEmONC* facility
Community 1 – Tertiary Hospital	180	31 (4 doctors, 17 midwives, 6 nurses, 4 CHWs)	CEmONC
Community 2 – Secondary Hospital	200	19 (2 doctors, 7 midwives, 2 nurses, 8 CHWs)	CEmONC
Community 3 – Rural Hospital	80	5 (2 midwives, 2 HEOs, 1 CHW)	BEmONC
Community 4 – Health Centre	39	24 (2 midwives, 1 HEO, 9 nurses, 12 CHWs)	N/A
Community 5 – Community health post	0	2 (1 nurse and 1 CHW)	N/A
Total	499	81	-

CEmONC – Comprehensive Emergency Obstetric and Newborn Care, BEmONC – Basic Emergency Obstetric and Newborn Care

### Study participants and recruitment

Community members were eligible to participate if they were aged 16 years or more, had experience with maternity care at a health facility as a parent, carer or relative, and resided in the catchment areas of one of the five participating facilities. With the assistance of community leaders, eligible women and men were recruited using a convenience approach and invited to participate through church, market and other community-based groups. Researchers (PM, RS, PH and DK) visited these settings, provided information about the study face-to-face with potential participants, and invited interested participants to take part. All participants that were approached agreed to take part. Discussions were conducted in each of the participating facility catchment areas.

### Data collection and management

Focus Group Discussions (FGDs) were led by PNG researchers (PM, RS, PH, DK) who live in East New Britain and have experience with local maternity care services. FGDs were preferred over one-to-one interviews, as FGDs promote interaction among participants and can generate deep and rich discussion about social and community norms [34, 35]. Prior to data collection, the research team undertook a two-week qualitative research workshop delivered in collaboration with the PNG Institute of Medical Research. The workshop provided an opportunity for upskilling in qualitative methods, refining and piloting focus group discussion guides with community members and exploring culturally respectful conduct of focus groups.

Ten FGDs (one female and one male FGD in the catchment area of each of the five participating communities) were conducted. Each FGD took between 1–2 hours to complete and were conducted by gender-concordant trained research officers using an interview guide to explore the following key topics: experiences of labour and birth care, thoughts on quality care, enablers and barriers to facility births, role of male partners in pregnancy and birth, and suggestions for improvement (full discussion guide available in the supplementary material 2 and 3). FGDs were conducted in community halls in each of the facility catchment areas (none were conducted in the study health facilities to reduce the risk of reporting bias). Groups were organised into male only and female only groups, based on advice from the local research team. Only researchers and participants were present during discussions and participants were not known to researchers. Participants provided both verbal and written informed consent prior to commencing discussions. Discussions were held in local languages, that is, Tok Pisin or Tok Ples or English (depending on group preference) and audio recorded. Whilst thematic saturation was reached after six FGDs, we continued sampling and conducting discussions to ensure we captured community perspectives serviced by different health facilities along the care pathway, from a remote community health post to a tertiary hospital. Discussions were transcribed verbatim into the language of the discussion and then translated into English to ensure accurate translation of meaning. Field notes were cross-checked with participants following FGDs and there was no further follow up with participants.

### Data analysis

Data were manually analysed using thematic analysis in NVivo, using the Braun and Clarke six step approach – i) familiarization with the data, ii) generation of codes, iii) identification of themes, iv) reviewing of themes, v) definition and naming of themes, and vi) producing the report. Initial coding was undertaken by five members of the study team (AW, RS, PH, PM, DK) (Australia and PNG) over a week in March 2020. The research team inductively coded the same transcripts by hand, constantly comparing coding and iteratively developing a code framework throughout the process. Whilst the researchers initially met face-to-face to conduct the coding and analysis, due to COVID-19 related border closures and travel restrictions, discussions continued using an online platform. The team met weekly over a three-month period where they discussed the transcripts, codes and continued to refine the code framework and themes.

The five domains relevant to the care experience in the WHO Quality and Maternal and Newborn Care framework [2, 3] were applied to the study findings to assist with interpretation and presentation of themes resulting in a combined inductive/deductive data analysis approach. The collaborative data analysis approach meant that Papua New Guinean researchers were central to determining the narrative and ensuring that the findings generated and interpreted reflected local views [36]. The findings were also discussed at a two-day Quality Maternal and Newborn Care Workshop held in East New Britain in November 2020, attended by 35 healthcare workers, managers and community members, providing an additional opportunity to member-check and validate study findings.

## Results

**Fig. 2:**
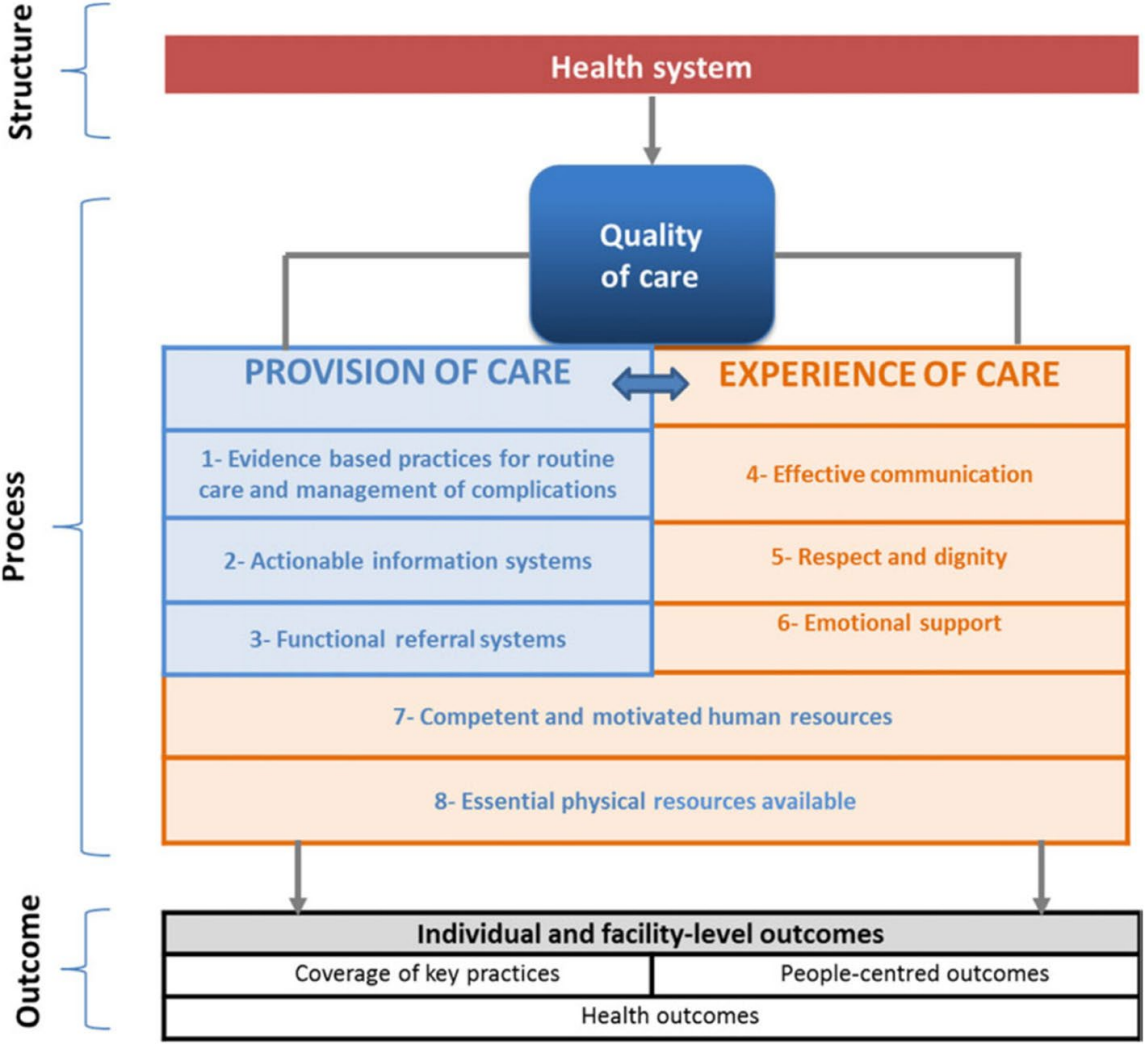
WHO Quality Maternal and Newborn Care framework experience domains *(in orange)* reproduced with permission from Tuncalp et al. 2015. [2]

A total of 32 women and 36 men participated in ten FGDs (FGDs ranged in size from 4 to 11 participants) (Table 2). Participants were parents and grandparents, ranging in age from 20 to 55 years. The findings are presented in accordance with the five experience of care domains (Fig. 2): i) effective communication; ii) respect and dignity; iii) emotional support, vi) competed, motivated human resources; and v) essential physical resources.

**Table 2:** Participant characteristics

Focus Group Facility Catchment	Number of participants – Women’s Focus Groups	Number of participants – Men’s Focus Groups
Community 1 – Nonga General Hospital (Tertiary Hospital)	5	5
Community 2 – St Mary’s/Vunapope Hospital (Secondary Hospital)	7	8
Community 3 – Kerevat Rural Hospital	11	9
Community 4 – Napapar Health Centre	6	6
Community 5 – Malasait community health post	7	4

### Effective communication

All participants spoke about the importance of good communication and the desire for more information about what would happen during labour and after birth and how to care for themselves and their baby. Communication from healthcare workers was described as limited with little explanation of what was happening. A common response was *“we don’t ask, we just listen and follow what they say”* [Woman, Community 5]. Some women also expressed a desire for greater information from healthcare workers about caring for their baby when they go home, *“the nurses need to give good advice on taking care of the baby regarding illnesses when they go home.”* [Woman, Community 2].

Men stressed the importance of healthcare workers keeping them informed about their partner’s health and progress during labour and birth. Men described being told to leave the labour ward, waiting anxiously outside and hearing their partner screaming and crying, but not knowing what was happening:

“*I didn’t know where the baby was. Then I went in and asked a nurse. I asked [the] sister and she told me that, ‘my baby had died and is at the nursery’. That was all I heard. So, I see that there is no communication of information at the health facility to us fathers. So there is a great need for us now that there must be communication, and it will be good for us to know what situation the women and children are in”* [Man, Community 3].

Communication barriers existed between health workers and women from villages outside the catchment area who spoke languages other than Tok Pisin (‘pidgin’) or English, with participants expressing discomfort when attending health facilities due to these communication challenges, “*Speaking in pidgin is a problem to some mothers here because they speak their own language and if you are talking to the mothers in the village they only speak their own language”* [Man, Community 4]. Not only was communication described as limited, but participants also described instances where they were scolded by healthcare workers:

“*We go to [facility], sometimes the staff don’t talk properly to them, they get cross because they don’t come at the right time when they are working. The men are scared to go with their wives because the staffs don’t talk properly to them like give them some good comments…That’s why they get scared because of that and so they don’t like to bring their wife to the hospital”* [Man, Community 5].

### Respect and preservation of dignity

Participants who were from outside the facility catchment area felt discriminated against and that they were discouraged from attending the facility. Participants described how they would observe healthcare workers ‘favour’ known friends and family, whilst women that were unknown to healthcare workers were more likely to be neglected or poorly treated:

“*When her [healthcare worker] best friend comes to [give] birth, they quickly attend to her. And if it is not their friend…they will punish the poor one like they don’t know her… they will leave her there feeling pain until they punish her, they will talk harshly to the poor mother. This is not the way, this favouritism is in the hospital with the doctors and the nurses up there. They say, ‘Who are you?’... they will pass by like they don’t know you”* [Woman, Community 4].

Participants spoke about how healthcare workers were often slow in providing care, leaving women and their partners feeling ignored and neglected. Long waiting times were a common experience. Women repeatedly spoke about how their feelings were denied, describing experiences where they could feel that the baby’s birth was imminent, shouting for help, and yet would be left ‘catching the baby’ on their own. Both men and women described instances of poor communication, where staff would shout at or mock women and use unfamiliar words:

“*They won’t treat you properly or they will be fast [angry] with you when you are screaming inside. They won’t attend quickly … they will scream at you. They will say, ‘are you a new mother?’ and sometimes when the mother is feeling pain and screaming, they will make fun of them…they use fancy Pidgin words too, the nurses”* [Woman, Community 4]

*“Most of the staff swear [at] the mothers when they are in pain, they [staff] will swear and say all types of word like, ‘you pussy’ or ‘be quiet’ or ‘you shut your mouth’”* [Man, Community 4].

While direct experience with maternal and perinatal death was commonly described across FGDs, openly speaking about the experience was less common. Men particularly spoke of wives, sisters, daughters and children who had died during pregnancy or childbirth. Some men mentioned that they had carried grief for a long time and had never spoken about it, “S*he [participant’s daughter] was in coma, she has heavy bleeding…she went, end of her life…I did not tell anyone about this till today I say it out loud”* [Man, Community 2].

### Emotional support

Women consistently said they wanted a support person present during labour and birth. They felt alone in hospital and were disappointed that they were not able to have support people present. Women identified many different options for support people including mothers, partners, aunties, sisters and friends. Women felt that the decision, about having a support person and who that could be, should be up to them, “*My daughter was crying wanting me to go with her into the labour ward and they said no mothers are to go inside. So my daughter kept on crying saying; “I want my mum to come inside…because it was her first time to have a baby”* [Woman, Community 1].

Men wanted to be present during labour and birth and said it was important to them. For example:

“*Yes! Yes! Yes! Because she is part of me and she is inside, I need to be with her to comfort her on what is happening to her. When I am outside, I am confused, will she be okay or not, for me outside I have no good thoughts. I am pressured walking back and forth in front of the gate. If the staff could just tell me to go inside so I can hold my wife’s hand and be with her. As a father I am willing to go into the birthing room one fine day to be with my wife, if the staff allows me to go inside”* [Man, Community 2].

Men described how they could support their partners, including providing food and water, physical support, pain relief, advocating for women and making up for staff gaps, especially when facilities were short-staffed. Being present during labour and birth were also described as potential opportunities to change attitudes around family sizes, *“Many of the men are fond of just having kids on and on because they have never been there, seeing the thing. If they see the mother in labour pains, ‘Ok! Ok! Mother was in pain so I must slowdown in having children’”* [Man, Community 1].

Despite wanting to be present, many men described multiple instances where they were told to leave the hospital and wait outside. Reasons provided by staff included that it was against hospital policy, or there was a lack of privacy in the birth suite, with multiple women labouring and giving birth in the one room:

“*So the nurse took my wife in the labour room…birthing room and she told me “Sorry son you will wait outside,” and I asked her “Why? She is my wife and I have to come in there with her,” but the nurse said, “There are plenty mothers who are in labour and it’s not good for the males to come inside.” So I just follow what the nurse said and I stayed outside”* [Man, Community 2].

Men noted that cultures and customs may also deter men from supporting their partners during pregnancy and birth, but others felt this could be overcome by normalising men being present and making it more common practice:

“*Maybe our culture does not allow men to go witness their wives giving birth. But I think it will be good for us fathers to be involved in childbirth as well. It will help with the bondage and relationship with our wives and children. I also believe that [the] father being present will make it more easy for other men to support the childbirth process”* [Man, Community 3]

### Competent, motivated human resources

Women and men spoke about the need for sufficient staff who are competent, well trained and kind. Women spoke about instances of supportive care and the difference this made to their experience, such as, when they were treated kindly, encouraged during labour and birth and provided with something to eat and drink. Both women and men emphasised the role of the healthcare workers in providing quality care and spoke about the need for healthcare workers to be thorough, ask the right questions, give information and advice and meet the needs and preferences of women:

“*The nurses must come and approach the mothers properly, talk nicely to them and tell them what to do or things like that. Sometimes when we are in need and we want to ask the nurses, the nurses are not there to help. We feel shy and scared to ask them for help”* [Woman, Community 2].

Participants spoke about how kind and caring healthcare workers could improve the overall experience of care:

“*They [healthcare workers] know that we need help and they must come and take us straight to the right place where this service must take place. The other thing is talking nicely, “are you alright?” greet us properly and then this will be comforting to us”* [Man, Community 3].

### Essential physical resources

Essential physical infrastructure was often lacking. Women and men spoke about the challenges of attending a health facility to give birth and once they got there finding the facility underequipped and under resourced. There were multiple barriers to accessing facilities including the distance between their village and facility, costs related to transport, facility fees, and language barriers. There is a lack of public transport options in East New Britain and most families do not own a vehicle, as such transport limitations were a major barrier, as well as the prohibitive cost of accessing private motor vehicles and other vehicles after hours and on weekends:

“*We find it so difficult with transport here because of the vehicle fees for mothers to go and get help or access the health facilities…We don’t have such cash crops like cocoa or coffee, we find little cash by selling crops like peanuts and taro. If your husband is working, then he will help you and if not you will have problems”* [Woman, Community 5].

Despite the challenges, women and men believed that the health facility was the best place to give birth, but they felt that many changes were needed to create an environment that was welcoming, safe, clean and private. Women talked about the need for a designated space for mothers and babies, where women had space and privacy to labour freely, to make noise and move around, away from hospital thoroughfares. The lack of privacy in facility birth suites left women feeling exposed and undignified, and prevented partners and other guardians from being present,

“*In the birthing room there is no privacy, it’s just open. Like, it should be a room for a mother. In the birthing room it’s just open and mothers can see each other. When the nurses come to help a mother to give birth and the other one is calling for help, there is no privacy”* [Woman, Community 2].

All participants spoke about the need for facilities to be cleaner with showers and toilets regularly cleaned, rubbish thrown out, bed linen cleaned and changed, spaces for women to dispose of blood-soaked pads after birth, “*They must clean the labour ward. Clean it properly so that it becomes comfortable and safe for the mothers to go in and birth”* [Woman, Community 1].

Participants spoke about how the local facilities were often short-staffed without essential equipment and medicines, which was a deterrent to giving birth there, “*For all this time now, I see mothers go for their clinic but when it comes to childbirth, they don’t come to the health facility. Because we don’t have the proper things to use on the mother… They say, ‘there are no good bed and no other instruments to use during the time of childbirth, so they go and give birth in the village’”* [Woman, Community 5]. Men also spoke about the need for facilities to have other basics available and functioning utilities, including water, electricity, medicines, equipment and training for staff, “*Infrastructure, health facilities must have medicines and everything that we need and want should be available…Electricity, water, they must be always there 24 h”* [Man, Community 3]. Generally, women are discharged 24 h after birth, however, allowing women to stay in the facility for longer periods was suggested to give women more time to recover and receive advice about how to care for the baby.

The physical environment and resources could be improved. Women and men spoke about the need for bigger and better beds; currently the beds were too narrow and uncomfortable. There was not enough room on the bed for mother and baby, and some women described incidents where babies had fallen off the bed. Women spoke about how they had needed something to eat and drink following birth, but nothing was available, relying on companions to bring and prepare food and drinks. Partners suggested that facilities provide women with food and talked about the cost and difficulty associated with preparing food, as they would need to go to the market and facilities generally lacked cooking facilities:

“*I want the health department to have all the health facilities with proper kitchens for mothers. Some their villages are away from the health facilities, or some are facing road conditions, when it rains and there is flood on the road the mother’s food would not come because of [a] flood and guardian is waiting for the vehicle and the poor mother is in hunger. So I want the health facilities to have proper kitchens which they can install power, electricity which they can boil water in the jug or even gas stove, electric stove. There must be a cook in the kitchen and they must make sure that every mother in the maternity ward must have breakfast, lunch and dinner”* [Man, Community 2].

Community members perceived the quality of care to be lacking in many facilities, especially in smaller, remote centres which were severely underequipped and understaffed. Challenges getting to facilities were also greatest for remote villages. Multiple areas for improvement were noted including better communication, facilitation of companionship, respect and privacy. The importance of health provider attitudes in improving the care experience and the importance of the facility-built environment in providing a private, clean and safe place to give birth were also seen as critical issues.

## Discussion

This study sought to understand women and men’s experiences and perspectives of quality maternal and newborn care in a rural community of PNG, as well as their suggestions for improvement. The community discussed the daily realities and challenges in accessing care and facilities that were under-staffed and under resourced. The importance of good communication and competent, caring healthcare workers was emphasized by participants. Both women and men expressed a strong desire for companionship during labour and birth. Community members also described simple changes that could immediately improve the quality of maternal and newborn care.

The daily realities of accessing maternity care in PNG – lack of transport, costs, insufficient health workforce, dilapidated facilities, unavailability of essential medicines and supplies, language barriers – were noted by participants. These challenges are not unique to East New Britain and have been reported in other studies conducted in PNG [7, 23, 28, 37, 38]. For example, a qualitative study with women from the Eastern Highlands of PNG similarly found geographical, financial and language barriers limited women’s ability to access health services [7].

Health worker attitudes that are interpreted as unwelcoming, unkind or inattentive can have a significant impact on the experiences of women and their partners, and influence whether a woman may attend a facility for birth [39]. A qualitative study with women in PNG similarly found that health worker attitudes that were rude and unfriendly, deterred women from giving birth in a health facility [7]. Similarly, studies in other LMICs have found that a lack of person-centred care, an unwelcoming reception on admission and a poor relationship and experience with healthcare workers significantly impacts the care experience and family willingness to attend facilities [40–47]. A positive relationship between the maternity provider and woman is highly valued and a key determinant of good quality care [48–50]. We have previously reported on how health providers in East New Britain want to provide quality maternity care [51, 52], but are constrained by a lack of education, training and health system limitations. Respectful care training is helpful [17, 53], yet the reality of working in an environment with a high work volume and insufficient staff and resources [54, 55] makes implementing respectful care challenging. In addition, whilst this research did not specifically explore the relationship between gender and quality maternal and newborn care in East New Britain, gendered attitudes and asymmetries of power that exist between health providers and women and between women and their partners can undermine intentions to provide quality care, especially respectful care [56]. We found that women wanted companionship in labour but often did not have a support person present, and many men wanted to be present to support their partner. There is limited research regarding women’s views on companionship during labour and birth in PNG or the small island nations of the Pacific. We have previously noted the desire of women and partners to have companions present during labour and birth in East New Britain, and the enablers and barriers to companionship [51]. Research from other LMICs (Egypt, Ghana, Iran, Kenya, Lebanon, Malawi, Syria and Tanzania) similarly found that women desired a support person during labour and birth although again, this was not always possible [49–54]. Companions can provide support to women in multiple ways, including emotional, physical and practical support, this may include reassurance and encouragement, physical touch such as warm baths/showers and massage, providing food and fluids, and advocating for the woman on her behalf, such as, requesting pain relief [55]. Research conducted worldwide has similarly identified multiple social, emotional and physical health benefits associated with companionship during labour and birth [57–61]. Health facilities should facilitate the presence of labour companions and respect women’s preferences regarding their choice of support person [62]. Initiatives to encourage and facilitate the presence of labour companions are also a low-cost, sustainable and effective way to improve women’s and their partners’ experience of care, particularly in facilities with a shortage of midwifery and nursing staff [63].

Men’s involvement in maternity care in PNG is limited and influenced by culture and customs [64–66]. We found that many men from communities in East New Britain wanted to be present during labour and birth, which is in contrast to exploratory research with men from the Southern Highlands of PNG, which found that men considered pregnancy, childbirth and child rearing to be women’s responsibilities, and that sociocultural norms and taboos were the most significant barrier to men’s involvement [66]. In this study, we found that men recognised that certain cultural values and social norms may limit men’s involvement, however obstructive hospital policies and health care providers that did not allow or encourage men to be present were identified as the main barrier. Women and men also acknowledged that the lack of privacy within birthing rooms was a barrier to companionship. Prohibitive hospital policies, healthcare providers and a lack of privacy have also been identified as barriers to companionship in East New Britain [51] and other LMIC settings [63, 67–69]. In almost every FGD, participants spoke of experiences where a sister, aunty or daughter had died during pregnancy, childbirth or in the postnatal period. Similarly, many spoke about newborn deaths. It is worth considering whether this familiarity with death generates a tolerance of poor quality of care, despite a wealth of evidence that many perinatal deaths are preventable, especially in LMICs [70–74].Yet familiarity with death does not equate to acceptance and it is important to note that participants, particularly men, spoke about how they had carried grief associated with a death for a long time. They expressed not speaking openly about these experiences and how they felt that it would be beneficial for health facilities to provide counselling services. These findings highlight the importance of seeking and listening to community and client experiences of quality care, in this case experiences of perinatal death. Quality care is important throughout the childbearing continuum, including during times of perinatal death, and there is a need for greater recognition and investment in perinatal death support services for parents in LMICs [75, 76], along with efforts to improve care seeking and health service access to avoid preventable perinatal deaths. A systematic review of parents’ experiences of stillbirth in LMICs found that a lack of recognition of stillbirth contributes to parental experiences of stigmatisation, blame, devaluation, and loss of social status [76].

Women and men reported that the health facility is the safest place to give birth, consistent with other studies in PNG [23, 38]. Yet given the challenges in accessing care, the under-resourced and -equipped facilities and the sub-optimal experience of care when present, it is important to consider how long the community will be willing to attend facilities for birth when their experience of care can be so poor. There is a need for major investments in the care experience, such as, empathetic and respectful care, the provision of food and water, comfortable bedding, clean facilities, and the presence of a support person. These changes would vastly improve the community’s experience of care and may encourage more women to attend facilities for antenatal care and birth [23]. The need to make facilities ‘more attractive and user-friendly’ has been previously reported by researchers in PNG as an urgent priority to increase facility-based birth rates [23]. Beyond increasing facility-based birth rates, improving respectful care as a key aspect of quality care is critical to improving health outcomes for mothers and babies [53].

### Strengths and limitations

This study has several strengths. We used a participatory approach whereby the study conception, design and implementation were co-developed with local stakeholders including the provincial health authority, health care providers and community members. FGDs were conducted by local, experienced research officers in local languages. As per community preferences, discussions were conducted separately for women and men to enable open discussion. Data analysis and interpretation were conducted with a research team from PNG and Australia, to ensure that findings reflected local narratives and perspectives and enabled reciprocal research capacity strengthening. It is important to note that the views represented are from community members and are not necessarily reflective of health worker actions or practices. We were not able to explore the experiences of care for women who are known to have poorer maternity care experiences, including young women, women with a disability and unmarried women [77, 78].

### Implications for policy and practice

These findings highlight that responsive, inclusive, and respectful care is not an optional extra [79], it is a key component of quality care and wanted by the community. Health facilities need to prioritise providing care that welcomes women and their support people, allows adequate privacy, and clean and comfortable spaces to labour and give birth. The facility birth rate in PNG is unlikely to improve until health services can meaningfully address these factors [23]. Small changes like the provision of food to birthing women, water and comfortable beds may make a big difference. However, the ongoing maintenance and repair of health facilities, competent and respectful staff, functional equipment and adequate medications and supplies are essential. The findings from this study have been used to inform the development of provincial quality improvement activities in East New Britain. Our findings are also relevant to the national PNG Government’s Maternal Health Taskforce, established in 2009 to address poor maternal and newborn health outcomes in PNG [80]. Moreover, these findings will be relevant to health services throughout PNG, and other Pacific nations, which face similar challenges to improving the quality of maternal and newborn care.

## Conclusion

Improving the quality of maternal and newborn care in PNG is a key element for improving maternal and newborn health outcomes. Community perspectives and experiences are essential to inform effective and sustainable interventions to improve the quality of maternal and newborn care and increase facility-based births. Community members expressed a desire for support people to be present during labor and birth, and for welcoming, clean and private birthing spaces. Poor experiences of care are significant disincentives to facility-based births and require special attention beyond infrastructure improvements and enhanced resources. A greater understanding of the care experience as a key component of quality care is needed and any quality improvement initiatives must include the user experience as a key outcome measure.

## Supplementary Information

**Additional file 1.**

**Additional file 2.**

**Additional file 3.**

## Data Availability

The datasets generated and/or analysed during the current study are not publicly available due to potential confidentiality concerns. Additional information can be made available from the corresponding author on reasonable request. Please contact the Scientific Integrity Officer (admin@burnet.edu.au) for more information.

## Abbreviation

PNG: Papua New Guinea
