# Supplementary material for: Community perspectives and experiences of quality maternal and newborn care in East New Britain, Papua New Guinea
Source: BMC Health Serv Res. 2023 Jul 20;23:780. doi: 10.1186/s12913-023-09723-x (PMC10360243; doi:10.1186/s12913-023-09723-x)
Supplement: Supplementary file 2 — Additional file 2. [file 12913_2023_9723_MOESM2_ESM.docx]

**Focus Group Discussion Guide – Community members (Women)**

**Introduction (Long statim)**

1. Tell us about yourselves (Go around the circle) [(Inap yupela wanwan stori long mipela long yupela yet (Raunim grup na askim)]
   - Name, age, where you live, how many kids you have, ethnic identity, religion etc (Askim long neim, krismas, ples nau em stap, hamaspela meri em gat, hamaspela pikinini em gat, liklik ples bilong em, lotu bilong em na kain olsem)

**Women’s experiences (Ol stori bilong wanwan meri)**

1. Would anyone like to share a story about their most recent birth? (Inap long wanpela long hia bai laik long serim stori bilong em long laspela beibi em karim)
   1. How did you make the decision that you were ready to give birth and which hospital did you go to? (Yu bin kamap wantaim dispela tingting olsem yu redi long karim olsem wanem na yu bin go long wanem hausik?) Was there a referral made? (Ol bin salim yu long narapela hausik kam/ol bin salim yu go long narapela hausik long karim?) What costs were involved and how did that affect your decision? (Hamas moni/ol narapela samting, i mekim yu long kamap wantaim dispela tingting long kam long dispela hausik?)
2. Tell me about what happened when you got to the hospital? (Stori long mi long ol wanem samting i bin kamap taim yu kam kamap long hausik?)
   - Who attended to you? (Husait i bin kam wok long yu?)
   - Were you examined? (Ol bin sekim yu?)
   - How long did you have to wait for a bed? (Yu weit hamaspela minit/awa bihain long ol wok lain givim yu bet?) Were you offered any food and/or water? (Ol bin givim yu wara na kaikai?)
3. Can you tell me about the labour ward/delivery room? (Inap yu stori long haus karim/rum bilong karim beibi?
   - Who was allowed to go into the ward/room? Why? (Husait bai inap long go insait long wod/rum?) Bilong wanem dispela ol lain tasol bai inap long go insait long wod/rum?
   - Who makes the decision about who can go into the room? (Husait i save mekim dispela tingting/disisen bilong husait i ken insait long rum?)
   - What privacy did you have? (Wanem kain ol samting i stap long mekim yu ino sem/poret long taim bilong karim?) How did this affect your delivery? (Dispela i bin mekim wanem long taim bilong karim?)
4. Tell me about who cared for you during your labour and birth? (Stori long mi long husait i bin lukautim yu long taim yu bin pilim pen na long taim bilong karim?)
   - What did they do for you? (Ol bin mekim wanem long yu?)
     1. Induction, pain relief, medical review, shower etc (Ol wok lain bin givim yu marasin long halivim yu long karim? Ol pen marasin? Ol wok lain sekim yu? Ol wok lain wasim yu?)
   - What type of support or encouragement did the staff provide? (Wanem kain ol sapot na toktok ol wok lain i bin givim yu long mekim yu pilim gut?)
   1. What did the healthcare worker say to you and your partner about what was happening during labour/childbirth? (Ol wok lain i bin tok wanem long yu na masta bilong yu taim yu wok long pilim pen? Na taim yu karim?) Were updates provided? (Ol wok lain i bin wok long toksave long yu long wanem samting i wok long kamap long yu long dispela taim?) Were you able to ask questions? (Yu bin pilim isi long askim ol askim long dispela taim tu?)
      1. Who was there at the birth? (Husait i bin stap long taim bilong karim?)
   - Can you tell me about the delivery of the baby? (Inap yu stori long mi long taim yu karim beibi.)
   1. Any emergencies, use of instruments? i.e. forceps/vacuum, blood transfusions, caesarean (I bin gat sampela bikpela hevi/birua kamap long taim bilong karim? Ol wok lain i bin usim sampela ol samting long halivim yu long karim? Kain olsem ol usim ol samting olsem spun/kap halivim beibi kam aut, givim bulut, katim bel bilong mama na kisim)
5. Can you tell me about what happened after your baby was born? (Inap yu stori long mi long wanem samting i bin kamap bihain long taim yu karim beibi?)
   1. What was done to you? (Ol mekim wanem samting long yu?)
      1. i.e. skin to skin contact, resuscitation, mother-baby separation, breastfeeding, perineal tear repair, mother cleaned, vital signs i.e. blood pressure etc, blood transfusion (ol putim beibi antap long bel bilong yu, ol givim win long pikinini, ol rausim beibi go long we long yu, givim susu, samapim rot bilong karim, klinim yu, sekim gut yu na beibi, givim bulut) (if mother and baby were separated, why?) (sapos ol rausim beibi long yu, bilong wanem ol mekim olsem?)
   2. What was done to the baby? Stori long wanem samting kamap long beibi?
      1. i.e. skin to skin contact, resuscitation, mother-baby separation, breastfeeding, immunisations or other medicines for the baby, baby bathed, cord care, eye ointment (ol putim beibi antap long bel bilong yu, ol givim win long beibi, ol rausim beibi go long we long yu, givim susu, ol givim marasin sut long beibi, wasim beibi, lukaut bilong beli baten, putim marasin long ai) (if mother and baby were separated, why?) (sapos ol rausim beibi long yu, bilong wanem ol mekim olsem?)
6. How was this birth the same or different to your other births? (Dispela karim i wankain/ino wankain long ol arapela karim bilong yu?) Better or worse? (Em orait o ino orait?)

**Perceptions about labour and childbirth at health facilities (Ol lukluk bilong ol meri long taim bilong pilim pen na long taim bilong karim pikinini long hausik)**

1. Thinking back over your experience, what things do you think should happen for all mothers and babies during labour and childbirth? (Wanem sampela ol samting yu ting i mas kamap long olgeta ol mama na beibi bilong ol long taim ol mama pilim pen na long taim bilong karim?) What things should be in place? (Wanem ol samting i mas i stap?) What should the experience be like? (Ol mama i mas pilim olsem wanem taim ol kam?)
2. What things do facilities need to work on to improve the care for mothers and babies? (Yu ting wanem ol samting hausik i mas wok moa long kamapim gutpela lukaut bilong ol mama na beibi?)
3. You have all spoken about your experiences with having a baby in a hospital, however, as we know not all mothers give birth in hospitals. (Yupela olgeta stori pinis long taim yupela kam karim beibi long hausik, tasol, mipela save olsem ino olgeta mama save kam karim long hausik.) What do you think are some of the reasons that affect a women’s decision to deliver at home or at hospital? (Yu ting wanem em ol sampela as/samting em mekim ol mama long karim long ples?) (i.e. cost, location, embarrassment, transportation, booked/unbooked, cultural, younger, past experience) Kain olsem; sait bilong moni, ples stap long we, sem long kam long hausik, rot bilong kam, skelim bel/ino skelim bel mama, tumbuna/kastom pasin, ol yangpela tumas, wanem ol lukim/pilim long pastaim ol kam)

**Knowledge of Quality Care**

1. What does quality maternal and newborn care mean to you? (Gutpela lukaut bilong mama na niupela beibi i minim wanem long yu?)

**Suggestions/Recommendations**

1. If you had the opportunity to speak to the health facilities about the things that make up good quality care – what are the top five things you would tell them? (Sapos yu bin gat sans long toktok wantaim ol hausik long wanem ol samting i kamapim gutpela lukaut, wanem ol faivpela nambawan samting yu bai inap long tokim?)

**(**One of the ROs to scribe top things listed on a sheet of paper**)**

1. Of these, vote for the most important thing first and then rank to the least important (Long olgeta dispela, makim wanem em impoten/bikpela stret i go daun long wanem em i liklik)

1. What are some other things you might like to say to the facilities about the quality of their care? (Wanem sampela ol arapela samting yu bai laik long tokim ol long sait bilong gutpela lukaut?)

### Closing

Thank you very much for your time and we will end here. (Tenkyu tru long taim bilong yupela na bai yumi bai pinis olsem.) We hope we can get the chance to talk to more community members about these things in the future. (Mipela hop long bai mipela gat sans long toktok moa long ol kominiti memba long ol dispela ol samting long bihain taim.)
