# Supplementary material for: Community perspectives and experiences of quality maternal and newborn care in East New Britain, Papua New Guinea
Source: BMC Health Serv Res. 2023 Jul 20;23:780. doi: 10.1186/s12913-023-09723-x (PMC10360243; doi:10.1186/s12913-023-09723-x)
Supplement: Supplementary file 3 — Additional file 3. [file 12913_2023_9723_MOESM3_ESM.docx]

**Focus Group Discussion Guide – Community members (Men)**

**Introduction (Long statim)**

1. Tell us about yourselves (Go around the circle) [(Inap yupela wanwan stori long mipela long yupela yet (Raunim grup na askim)]
   - Name, age, where you live, how many wives you have, how many kids you have, ethnic identity, religion etc. (Askim long neim, krismas, ples nau em stap, hamaspela meri em gat, hamaspela pikinini em gat, liklik ples bilong em, lotu bilong em na kain olsem)

**Personal experience (Ol stori bilong wanwan long sait bilong hausik)**

1. Are there a few people here that might like to share a story about when your wife, sister or someone you know gave birth in a facility? (Igat sampela ol lain long hia husait i laik stori liklik long taim meri/susa o wanpela meri yu save i bin go karim beibi hausik?)
   - Can you tell us about what happened? (Inap yu stori long wanem samting i bin kamap?)
   - How was the decision made to go to the hospital and which one did you go to? (Yu bin kamap wantaim dispela tingting olsem meri bilong yu i redi long karim pikinini olsem wanem na yu bin kisim em igo long hausik?) What costs were involved and how did that affect the decision? (Hamas moni/narapela samting i mekim yu long kamap wantaim dispela tingting long kisim meri/susa o wanpela yu save long kam long hausik?)
   - To what extent are you happy with how you were able to support your wife/sister during labour and childbirth? (Igo inap long wanem mak stret yu pilim hamamas long wanem ol halivim yu bin inap/isi long givim long meri/susa o wanpela yu save long taim em pilim pen na long taim em karim?) How would you like it to have been different? (Yu bai laikim dispela ol halivim yu bin givim i narapela kain olsem wanem?)
2. What were the barriers and facilitators to you being involved with the birth? (Stori long wanem ol sampela samting i bin mekim hat long yu long stap wantaim meri/susa o wanpela yu save long karim?) Wanem ol samting i bin mekim yu i laik stap long taim meri/susa o wanpela yu save long karim?)

**Men’s perception on maternal and newborn care in a health facility (Ol lukluk na tingting bilong ol man long lukaut bilong mama na niupela beibi insait long hausik)**

1. Tell me about what you think care at facilities should be like for mothers and their babies during labour and childbirth. (Stori long mi long wanem yu ting lukaut bilong ol mama na beibi bilong ol long hausik i mas olsem wanem long taim ol pilim pen na long taim bilong karim?)
   - Tell me about what resources i.e. medicines, equipment etc should be in place (Stori long wanem ol samting yu ting i mas i stap; kain olsem ol marasin, masin na ol samting bilong usim long wok)
   - Tell me what the room should be like i.e. privacy, cleanliness etc (Stori long mi long wanem yu ting rum bilong mama long stap mas olsem wanem; kain olsem long sait bilong ol mama long noken pilim sem/poret, sait bilong pipia na lukluk bilong rum)
   - Tell me what the health workers should be like i.e. how should they communicate with women and their families? (Stori long mi long wanem yu ting ol wok lain bilong hausik i mas mekim wanem? Kain olsem long toktok wantaim ol mama na femili bilong ol) How should they provide support to woman? (Yu ting ol wok lain i mas givim sapot long ol mama olsem wanem?)
2. Why do you think these things are important? (Yu ting bilong wanem dispela ol samting em I impoten/bikpela samting?)

**Enablers and barriers to health facility births (Ol samting mekim isi/hat long kam karim beibi long hausik)**

1. Some of you have spoken about the experience of your wife/sister having a baby in a hospital, however, as we know many mothers still give birth in the village. (Sampela bilong yupela i bin stori pinis long stori bilong meri/susa o wanpela yu save i bin kam karim pinis long hausik, tasol, mipela save olsem planti ol mama i wok long karim beibi yet long ples.) What do you think are some of the reasons that affect a women’s decision to deliver at home or at a hospital? (Yu ting wanem em ol sampela as/samting em mekim ol mama long karim long ples?) (i.e. cost, location, embarrassment, transportation, booked/unbooked, cultural, younger, past experience) Kain olsem; sait bilong moni, ples stap long we, sem long kam long hausik, rot bilong kam, skelim bel/ino skelim bel mama, tumbuna/kastom pasin, ol yangpela tumas, wanem ol lukim/pilim long pastaim ol kam)
2. Tell me about what you think is important to women when they come to the clinic/hospital for health care? (Stori long mi long wanem yu ting em impoten/bikpela samting long ol meri taim ol kam long hausik long kisim lukaut bilong hausik?)
3. Tell me about how you think women would want to be treated when they come to a health facility? (Stori long mi long wanem yu ting ol meri bai laikim ol wok lain long hausik i mas tritim/sowim wanem kain pasin taim ol kam long hausik?) What is the current practice? (Nau yet, em olsem wanem?)

**Men’s involvement**

1. To what extent do men support their wives during labour and childbirth? (Halivim na sapot bilong ol man long ol meri bilong ol i save i go inap long wanem mak long taim ol meri pilim pen na long taim bilong karim?)
2. What are the things that stops men from being involved? (Wanem sampela ol samting i save stopim/banisim ol man long ol ino ken stap tumas long dispela taim meri pilim pen na long taim bilong karim?) At the facility level? (Insait long hausik?) Community level? (Insait long ples?) Family level? (Insait long femili?)
3. What are the things that help men to be involved? (Wanem ol samting i save halivim ol man long stap long taim ol mama pilim pen na long taim bilong karim?) At the facility level? (Insait long hausik?) Community level? (Insait long ples?) Family level? (Insait long femili?)
4. What benefits could there be for women if men are more involved during pregnancy and childbirth? (Wanem em ol gutpela samting bai inap long kamap long ol meri sapos ol man i stap long taim ol mama bel na long taim ol karim pikinini?) What are the benefits for men if they were involved more? (Wanem sampela ol gutpela samting sapos ol man i stap moa long dispela kain ol taim?)
5. How important is the role of fathers in the quality of care for mothers and babies during labour and childbirth? (Wanem wok mak ol papa inap long mekim long kamapim gutpela lukaut bilong ol mama na beibi long taim mama pilim pen na long taim bilong karim em impoten olsem wanem?)

**Knowledge of Quality Care (Save bilong wanwan long sait bilong gutpela lukaut)**

1. What does quality maternal and newborn care mean to you? (Gutpela lukaut bilong mama na niupela beibi i minim wanem long yu?)

**Suggestions/Recommendations (Sampela ol tingting/toktok bilong mekim senis)**

1. What things do you think should happen for all mothers and babies during labour and childbirth? (Wanem sampela ol samting yu ting i mas kamap long olgeta ol mama na beibi bilong ol long taim ol mama pilim pen na long taim bilong karim?) What things should be in place? (Wanem ol samting i mas i stap?) What should be the experience like? (Ol mama i mas pilim olsem wanem taim ol kam?)
2. If you had the opportunity to speak to the health facilities about the things that make up good quality care – what are the top five things you would tell them? (Sapos yu bin gat sans long toktok wantaim hausik long wanem ol samting i kamapim gutpela lukaut, wanem ol faivpela nambawan samting bai yu inap long tokim?)

**(**One of the ROs to scribe top things listed on a sheet of paper**)**

- - Of these, vote for the most important thing first and then rank to the least important (Long olgeta dispela, makim wanem em impoten/bikpela stret i go daun long wanem em i liklik)
  - What are some other things you might like to say to the facilities about the quality of their care? (Wanem sampela ol samting yu bai inap long toktok long hausik long sait bilong gutpela lukaut?)

### Closing

Thank you very much for your time and we will end here. (Tenkyu tru long taim bilong yupela na bai yumi pinis olsem.)

We hope we can get the chance to talk to more community members about these things in the future. (Mipela hop long bai mipela gat sans long toktok moa long ol kominiti memba long ol dispela ol samting long bihain taim.)
